# Oxford-AstraZeneca Coronavirus Disease-2019 Vaccine-Induced Immune Thrombocytopenia on Day Two

**DOI:** 10.1155/2021/2580832

**Published:** 2021-07-21

**Authors:** Azhar Kareem Razzaq, Ameer Al-Jasim

**Affiliations:** ^1^College of Medicine-University of Basrah, Department of Haematology, Baghdad, Iraq; ^2^College of Medicine-University of Baghdad, Baghdad, Iraq

## Abstract

**Introduction:**

Vaccines have been one of the most impactful human discoveries that have significantly changed life expectancy. Immune thrombocytopenic purpura (ITP) is an autoimmune disease characterized by platelet damage, life-threatening thrombocytopenia, and haemorrhage when the platelet count reaches below 20 × 10^9^/mcL. Its pathogenesis involves viral mimicry or T-cell-induced immune destruction in antibody-negative cases. The clinical manifestations of thrombocytopenia vary according to the severity (level of platelets) and range from being asymptomatic to severe haemorrhage. ITP is treated with immunosuppression. *Case Presentation*. A 26-year-old Iraqi male laboratory analyst with an unremarkable medical history presented with severe thrombocytopenia 2 days after receiving the Oxford-AstraZeneca coronavirus disease-2019 vaccine. The patient was asymptomatic with unremarkable examination findings. However, his low platelet count was discovered accidentally, and the patient did not exhibit the resistance pattern of ITP and recovered successfully with regular immunosuppressant treatment.

**Conclusion:**

Patients with a history of thrombocytopenia can develop vaccine-induced thrombocytopenia earlier than the expected onset. Close monitoring, through regular complete blood counts, is highly recommended for patients with previous thrombocytopenia because the immune modulation process of the vaccine can worsen preexisting thrombocytopenia.

## 1. Introduction

Vaccines have become an impactful human discovery that has significantly changed life expectancy and disease incidence. However, adverse events and autoimmune phenomena, including immune thrombocytopenic purpura (ITP), have been reported in vaccination [1].

ITP is an autoimmune disease characterized by platelet damage that causes life-threatening thrombocytopenia through haemorrhage, when the platelet count drops below 20 × 10^9^/mcL. The pathogenesis of vaccine-associated autoimmunity most likely involves virally induced molecular mimicry or a T-cell immune-mediated mechanism [2, 3].

The clinical manifestations of ITP range from asymptomatic to light purpuric rash all over the body to severe intracranial haemorrhage [4]. The usual treatments for such conditions are immunosuppressants, such as steroids and intravenous immunoglobulin (IVIG) [2].

Different vaccines have reportedly caused ITP through various mechanisms, eventually leading to thrombocytopenia. Thus, it is not unusual for a vaccine to cause ITP [1]. On the 8th of June 2021, Welsh et al. reported the occurrence of immune thrombocytopenia with the Pfizer and Moderna vaccines in their manuscript regarding the COVID-19 Vaccine Adverse Event Reporting System. We report the first case of immune thrombocytopenia induced by the Oxford-AstraZeneca coronavirus disease-2019 (COVID-19) vaccine [5].

In this case, we present an Oxford-AstraZeneca COVID-19 vaccine-induced immune thrombocytopenia that occurred on the second day in a 26-year-old man with a medical history of undiagnosed thrombocytopenia.

## 2. Case Presentation

A 26-year-old Iraqi male laboratory analyst presented with severe thrombocytopenia two days after receiving the Oxford-AstraZeneca COVID-19 vaccine. He had an unremarkable medical history, except for mild thrombocytopenia, ranging from 100 to 130 × 10^9^. He had not sought consult for a provisional diagnosis. In addition to experiencing mild headaches, the patient had generally been asymptomatic. His low platelet counts were discovered incidentally through a routine complete blood count.

On examination, the patient was well, conscious, oriented, not in distress, and vitally stable. There was no gum or conjunctival bleeding, epistaxis, petechiae, purpura, or ecchymosis. Jaundice, organomegaly, and lymphadenopathy were also not noted.

Further laboratory testing revealed a platelet count of 64 × 10^9^/L on day 2, which dropped to 35 × 10^9^/L on day 3. The rest of his complete blood count results were unremarkable. His fibrinogen level was 145 mg/mL, and his prothrombin time, partial thromboplastin time, D-dimer, brain magnetic resonance imaging, and magnetic resonance venography were all normal.

The management plan consisted of hospital admission, methylprednisolone (pulse dose at 1 g/kg for 3 days), and IVIG (1 g/kg for 2 days).

The patient responded well to treatment and had a favourable outcome. On the fifth day, he had a platelet count of 90 × 10^9^ L with a normal coagulation profile. His platelet count normalized in the second week. The patient was then discharged based on his improved blood count and was recommended for periodic follow-up.

## 3. Discussion

There have been at least 25 reports of ITP related to the COVID-19 vaccine. Reportedly, only the Pfizer and Moderna vaccines caused this adverse event. However, this side effect is not exclusively attributable to these vaccines [5–8].

Thrombocytopenia is not an unusual outcome of an immune modulation process, such as vaccination. It is expected to develop 4–30 days after vaccine administration [9]. In the present case, severe asymptomatic thrombocytopenia was noted 2 days after the Oxford-AstraZeneca COVID-19 vaccine had been administered. This contradicted the known onset and symptomatology of vaccine-induced thrombocytopenia. This deviation was attributed to the patient's undiagnosed thrombocytopenia.

The patient's good clinical response to the standard ITP regimen confirmed the immune-induced process of thrombocytopenia. This was most likely associated with the corresponding vaccine. The clinical scenario was similar to that of patients who received the Moderna and Pfizer vaccines [5, 8].

The deleterious effects of aggressive immunosuppression on vaccine-induced thrombocytopenia should not be overlooked. In prior studies, postvaccination immunosuppressive therapy attenuated the immune response to the administered vaccine [10].

## 4. Conclusion

In conclusion, patients with a history of thrombocytopenia can develop vaccine-induced thrombocytopenia earlier than the expected onset. Further studies are warranted to investigate this phenomenon.

Close monitoring of patients with previous thrombocytopenia is highly recommended because the immune modulation process of the vaccine can worsen the preexisting thrombocytopenia. Thus, regular complete blood count is indicated after COVID-19 vaccination.

## Data Availability

The data used to support the findings of this study are available as gray literature (offline) and are available from the corresponding author upon request.
